# Acute Nephritis in the Army: A Curious Outbreak in Flanders

**Published:** 1915-12-11

**Authors:** 


### ACUTE NEPHRITIS IN THE ARMY.

### A Curious Outbreak in Flanders.

Some five or six months ago a curious outbreak of acute nephritis amongst our troops in Flanders was reported in the Press, and apparently some fears were entertained that a large number of cases might be about to arise. The whole subject of this outbreak and its causation was tackled at once in earnest by the authorities of the R.A.M.C.; and, so far as can be judged from the meagre information since published, the epidemic was effectually squashed before it had developed into anything serious. There were, according to statements which have been published,* some unusual and interesting features about this nephritis; and, although the Army medical authorities have very rightly practised much reserve on the matter, some of these features can be commented on without indiscretion.

### Epidemic Nephritis.

Soldiers, otherwise in good health, were attacked by the symptoms of acute nephritis of mild or moderate grade. That is to say, headache, œdema of the face and feet, nocturnal dyspnœa, reduction of the quantity of urine with a considerable percentage of albumin in it, were proportionally common; whereas uræmic convulsions, blood in the urine, vomiting, amblyopia, and other signs of severe toxæmia were seldom met with. Another interesting point was that, fortunately, all the symptoms subsided very rapidly indeed under simple treatment: far more uniformly, quickly, and satisfactorily than in the ordinary acute nephritis of civil life, whether scarlatinal or "idiopathic." Indeed, after a week or a fortnight the patients were, in the vast majority of cases, to all appearance perfectly well, with their kidneys functioning quite normally. Whether the temporary inflammation which subsided so quickly had passed off without leaving behind any permanent damage whatever, it is perhaps too early to say: certainly it seemed so at the time to those medical officers who had experience of these cases. At the same time, it was noticed that a proportion of the sufferers had distinct evidence of pre-existing arterio-sclerosis; such cases one would hardly expect to recover completely.

### Hypothetical Explanations.

When the epidemic was first noticed various hypotheses were put forward to account for it. One was that the Germans were poisoning the rivers and water supplies with some metallic poison. This hypothesis was tested, it is said, by careful analyses of water supplies and of the urine excreted by nephritis patients; and was disproved. Alternatively it was propounded that metallic contamination from water-carts might have occurred. The next explanation suggested was that the concentrated proteid of "bully beef" was proving too much for the metabolism of the soldier, and was leading to a small percentage of breakdowns in the renal structure and function. Against this it was counter-advanced that the troops have fresh—or, at least, frozen—meat whenever it is practicable to give it them, not bully beef; and that in South Africa, where bully was practically the sole proteid diet of the soldier, these cases of nephritis were not often met with. Then it was urged that, although bully beef alone might not account for the nephritis, yet bully beef, *plus* the exposure of trench life, might be at the bottom of the trouble. Yet again, in rebuttal, it was pointed out that in the winter nephritis was hardly ever seen, whereas the outbreak in question took place in the summer. Finally, some ingenious persons put forward what may be called the zymotic hypothesis: to wit, that the nephritis was merely an incident in a mild but unrecognised infectious fever, and that this fever might be either scarlet fever or enteric. Scarlet fever, as everyone knows, is always liable to be complicated by acute nephritis, usually a week or two after the fever has subsided: careful inquiries failed to elicit from the patients anything that could be construed as an abortive or mild attack of scarlet fever. That enteric fever may begin as an acute nephritis is mentioned in every text-book of medicine; but this form of onset is exceedingly rare, and as a matter of fact no confirmatory evidence has been obtained, so far as we know, of any enteric infection in these cases of acute nephritis.

It has been stated above that convulsions and full-blown uræmia were rarely met with in the epidemic. They were not, however, completely absent; for a small number of patients did display them, and it is said that there were even occasional deaths. Those who dealt with the cases seem to be convinced, however, that even the cases of pronounced uræmia coma, with hæmaturia or even suppression of urine, responded so wonderfully to simple treatment (hot packs, pilocarpine, venesection, lumbar puncture, calomel, and fluid diet) that in a comparatively short time they were fully convalescent. To explain how the Army Medical Service authorities dealt with this outbreak might perhaps be injudicious at the present moment; but, according to reliable accounts, it may be accepted that the measures were both prompt and effectual. Doubtless in due course, probably after peace is signed, an official report on the matter will be published. Until then comment is inadvisable, but the public may rest assured that the whole question was dealt with on broad lines, and that the issue was one on which all those concerned are to be congratulated.

[Since this article was accepted for publication a long account has appeared in the *Journal of the Royal Army Medical Corps* of the epidemic and of researches carried out upon its origin. As the conclusions arrived at are very similar to those independently reached by our contributor, we have thought it well to leave the latter just as they reached us.—Ed. The Hospital.]

* Notably in the *British Medical Journal*.

